# Modic changes and their role in vertebrogenic back pain: a literature review

**DOI:** 10.1007/s00256-025-05065-3

**Published:** 2025-11-03

**Authors:** Jimmy Wen, Megan Kou, Ihab Abed, David Park, Zohaer Muttalib, Arsh Alam, Foad Elahi

**Affiliations:** 1 California Northstate University College of Medicine, 9700 W Taron Dr, Elk Grove, CA 95757 USA; 2 University of California, Davis, 1 Shields Ave, Davis, CA 95616 USA; 3 California Center of Pain Medicine & Rehabilitation, 4944 Sunrise Blvd, Fair Oaks, CA 95628 USA

**Keywords:** Modic changes, Vertebrogenic back pain, Low back pain, Chronic low back pain, Basivertebral nerve, MRI

## Abstract

Vertebrogenic back pain (VBP) has emerged as a potentially undiagnosed subtype of chronic low back pain that is thought to arise from structural damage and subsequent inflammatory changes in the vertebral endplates and bone marrow. Modic changes (MCs) found on MRI have been proposed to be a potential indicator for VBP. However, the pathophysiology and clinical basis for MCs are still being investigated. This literature review aims to comprehensively synthesize the available evidence on the pathogenesis, etiologies, and therapeutic outcomes associated with MCs. MCs are classified into three types based on MRI characteristics, with the potential to present with mixed types or to interconvert over time. Risk factors and hypotheses include mechanical disruption, inflammation, bacterial, and autoimmune etiologies increasingly linked to nociceptive signaling from the basivertebral nerve (BVN), causing VBP. Diagnostic and criteria standardization is a major gap for further research to produce more consistent therapeutic outcomes. Future directions with biomarkers, advanced emerging imaging techniques, and clinical translation are required to refine the clinical role of MCs in diagnosing and managing VBP.

## Introduction

Chronic low back pain (CLBP) is defined as low back pain (LBP) that lasts for more than six months and is one of the most common causes of disability, missed work, and usage of healthcare resources worldwide [1]. The annual cost in the USA is estimated to be greater than $100 billion, with approximately 50 million physician visits [2]. However, the etiology of LBP remains broad and nonspecific, with causes ranging from genetics, smoking, obesity, advanced age, trauma, and psychosocial triggers [2]. Without a standardized diagnostic reference, many patients (80–90%) are diagnosed with nonspecific LBP, which leads to confusion in treatment modalities, causing mixed outcomes [1]. The multifactorial nature of LBP can result from the facet joints, ligaments, intervertebral discs, vertebral bodies, and spinal nerve roots, which require targeted interventions to optimize clinical outcomes for each [3].

Recent research has suggested that vertebral endplate changes significantly contribute to disc degeneration and transmit pain signals via the basivertebral nerve (BVN), leading to vertebrogenic back pain (VBP) [1]. The damaged endplates lead to the release of inflammatory markers and inflammation seen as Modic changes (MCs) in magnetic resonance imaging (MRI). Cytokines and osteoclastic factors play a major role in the development of MCs by regulating bone marrow and bone mass composition, which share characteristics similar to those of osteoarthritis changes [4]. Endplate defects have been proposed to be an important pain-generating source due to their proximity to the disc, and this communication can lead to a persistent inflammatory state, leading to pain [4]. Immunohistochemical analysis also found a high density of nociceptors (containing substance P, CGRP, PGP 9.5 nerve fibers) that travel to the BVN, which was greater in damaged endplates compared to normal endplates [4, 5]. MCs can be classified into types 1, 2, or 3.

MCs may be associative, predictive, or incidental findings in VBP. In general, they have been found to have a strong correlation with back pain [1]. However, it remains unclear whether MCs are directly responsible for vertebrogenic pain and the nociceptive signals originating from the BVN. The clinical relevance of MCs has been heavily debated over the years, and a consensus regarding their clinical significance has not been reached. Thus, this literature review aims to elucidate the current literature on the pathophysiology and etiologies of MCs, imaging characteristics, treatment options, and discuss the clinical implications of MCs, focusing on their role in VBP.

## Modic changes pathophysiology and anatomy

### Vertebral body anatomy

The superior and inferior portions of the vertebral body are covered by cartilaginous endplates that interface with the intervertebral disc [6]. These end-plates, like other cartilaginous structures of the body, are typically avascular in adults [6]. The avascular disc, with its sparse blood supply, relies on nutrient diffusion from the vertebral endplates [7]. MCs are highly associated with endplate changes in the adjacent vertebrae [8]. The blood supply of the vertebral body arises from the segmental arteries of the aorta. These arteries give rise to nutrient arteries that surround the vertebral body and enter through foramina, and course to form an intraosseous network to supply the trabecular bone [9]. The BVN enters the vertebral body through the basivertebral foramen, which branches off to supply the superior and inferior endplates. These endplates transmit nociceptive signalling following disruption or inflammation in this area [10]. Nociceptors and vertebral capillaries found in vertebral bodies can be anatomically traced back to the BVN [11]. A protein gene product 9.5 (PGP 9.5) staining study found that BVNs are CGRP-positive, showcasing their role in nociception [12].

## Pathophysiology

### MRI classification

**Fig. 1:**
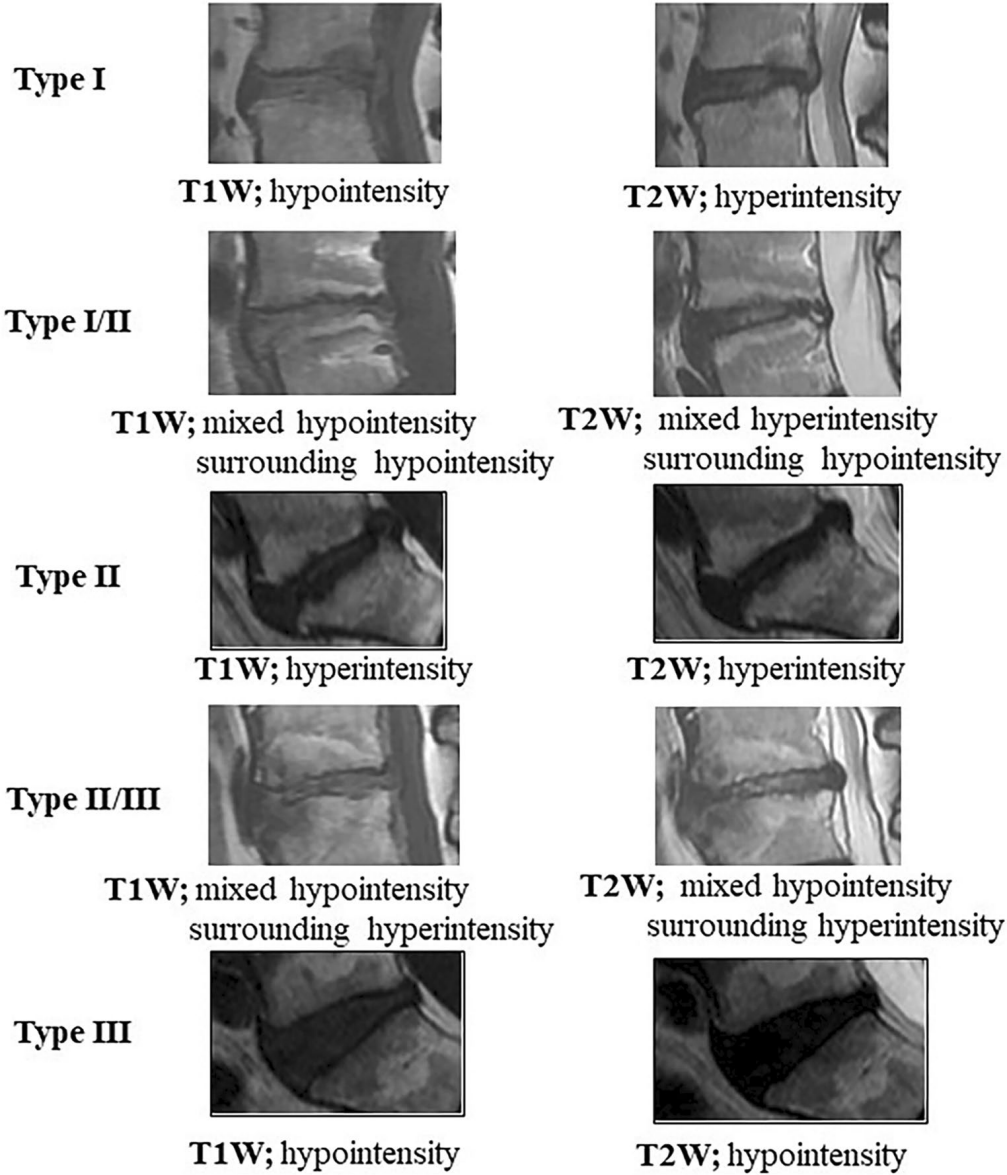
MRI examples of Modic changes types 1, 2, and 3 and subtypes. Reproduced from Teraguchi et al. [15], licensed under CC BY-NC 4.0

Disruption of this blood supply and degenerative changes can lead to nutrient disruption and inflammatory changes surrounding these structures, which likely play a role in fatty and fibrotic changes seen in MCs [13]. These changes in signal intensity are a result of structural changes following varying proposed theories. MC1s typically contain hypointense findings on T1W and hyperintense findings on T2W, indicating fibrous tissue replacement of bone marrow with trabecular thickening and granulosis, suggestive of edema and inflammation [14]. MC2s show hyperintense changes on T1W and range from hyper- to iso-intense on T2W, suggesting fatty replacement of marrow in addition to inflammation and edema as seen in MC1s [14]. MC3 are hypointense on both T1W and T2W and suggest subchondral sclerosis [14]. An example of the different types of MCs can be found in Fig. 1.

These sequences of findings for each type have varying implications when correlated with histologic findings of the lesions. Further classification, recently demonstrated by Rajasekaran et al., suggests expanding the MC framework into Disc End Plate Bone Marrow Complex, which, alongside further criteria, utilizes short tau inversion recovery (STIR) in addition to T1W and T2W imaging to classify types A–D. These criteria require further study and utilization to assess their clinical efficacy (Table 1) [15].

**Table 1:** MRI, histology, and pain correlation for types 1, 2, and 3 Modic changes

Type	MRI appearance (T1W/T2W)	Histology	Pain correlation
Type 1 MC	Hypointense/hyperintense	Fibrous tissue replacement of bone marrow	Highly correlated
Type 2 MC	Hyperintense/isointense to hyperintense	Fatty marrow replacement of bone marrow	Variable
Type 3 MC	Hypointense/hypointense	Subchondral sclerosis	Minimal
Disc end plate bone marrow complex classification			
Type		MRI appearance (T1W/T2W/STIR)	
Type A		T1W hypointense/T2W hyperintense/STIR hyperintense	
Type B		Hyperintense in all	
Type C		T1W hyperintense/T2W hyperintense/STIR hypointense	
Type D		Hypointense in all	

### Immunogenic mechanisms

Due to the lack of a significant blood supply, intervertebral discs, including their nucleus pulposus (NP) component, are immune-privileged sites. The immune system, encountering the cells of the NP, can trigger an immunogenic response that can result in inflammatory changes [16]. Dudli et al. suggest that the changes leading to MCs in this autoimmune cascade require an additional pro-inflammatory milieu. Specifically, IL-1α is required to induce significant upregulation of IL-1, IL-6, IL-10, and tropomyosin receptor kinase A and drive inflammation for the appearance of MCs [17]. Damage to this area in processes such as degenerative disc disease can lead to increased vascularity and thus exposure triggering immunogenicity and an inflammatory cascade [9].

### Traumatic etiologies

Following trauma to the spine through actions such as heavy lifting and motor vehicle accidents, compressive forces can cause microfractures and disruption of end-plate integrity. The subsequent inflammatory response, along with nerve and blood supply remodeling, can contribute to the development of MCs [14]. Chronic causes of stress/overload appear to disrupt the healing process and are less likely to resolve, whereas acute trauma can heal following removal of the initial insult [14]. MCs adjacent to degenerated discs resemble bone marrow lesions in the femur and tibia of osteoarthritic knees, both in their contribution to pain from joint degeneration and in their shared risk factors such as age, sex, and body weight [14]. This connection underscores the potential for research on osteoarthritis and bone marrow lesions to shed light on the pathogenesis of LBP caused by MCs.

### Bacterial contribution

**Fig. 2:**
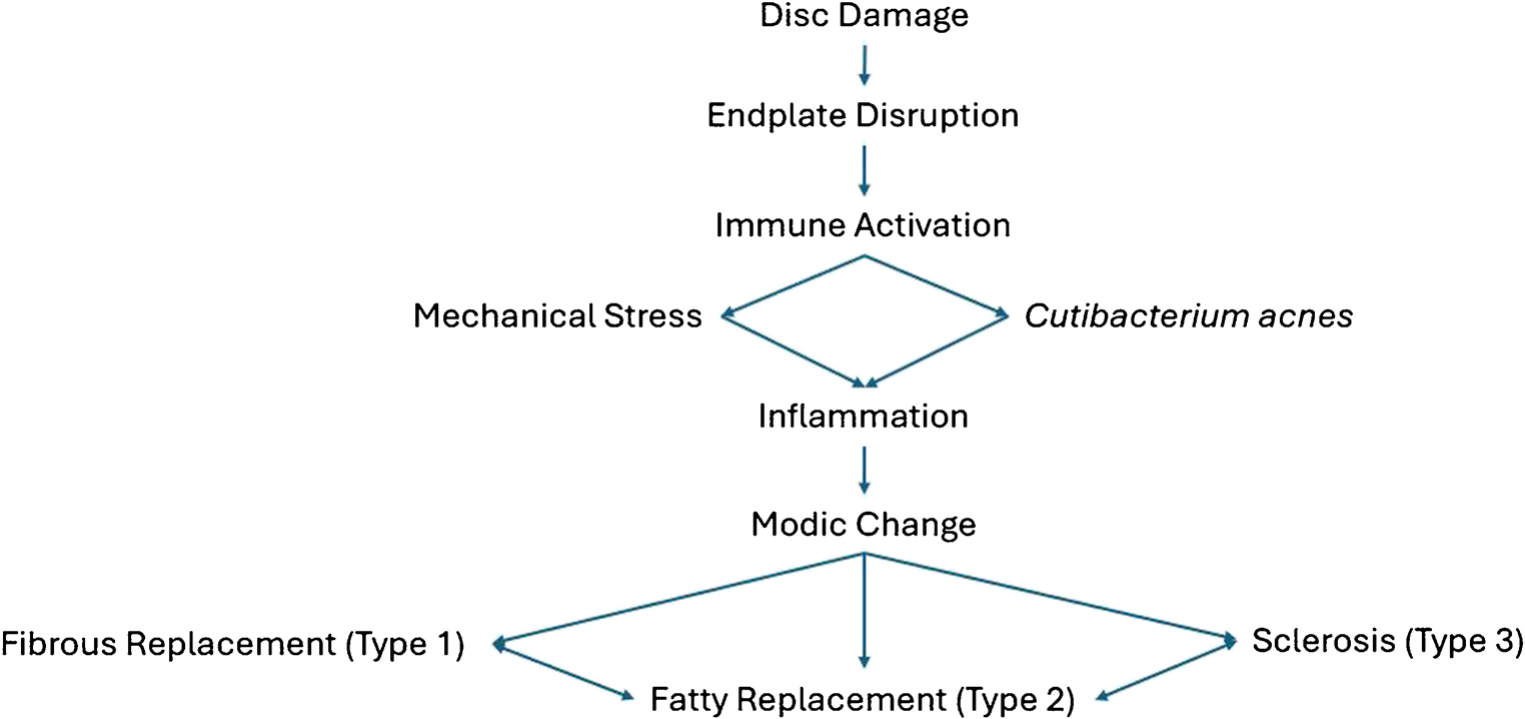
Pathophysiology of Modic changes

Following disc damage, the environment may be susceptible to contact and colonization by anaerobic bacteria and macrophage recruitment, resulting in compositional changes in the surrounding structures as seen in MCs following macrophage interaction [16, 18]. Large end plate changes trigger a series of degenerative interactions between the disc and vertebra, leading to elevated intraosseous pressure, impaired metabolite exchange, and promoting the migration of inflammatory mediators from the disc into the bone marrow, contributing to the development of lesions near the end plate [18]. In combination, the increases in cell death following endplate damage, with subsequent matrix breakdown, immunogenicity, and bacterial colonization, are thought to result in immune complex deposition, resulting in complement activation leading to MC1 and MC2 [19]. This pathway can be visualized in Fig. 2.

## Bacterial or autoimmune etiology

MCs have been associated with low-grade bacterial colonization or autoimmune responses induced by accumulated endplate damage [19]. These mechanisms lead to focal endplate and marrow inflammation that leads to edema and signal changes on MRI. Both processes occur without the disc destruction, collapse, or soft tissue involvement seen in discitis/osteomyelitis (Table 2) [20]. *Cutibacterium acnes* (*C.acnes*) has been found in excised intervertebral disc [20]. Additionally, when injected into rat tail intervertebral discs, *C.acnes* leads to endplate resorption and MC1-like changes under MRI [21]. Braten et al. investigated the effect of Amoxicillin in patients with CLBP and MC 1 or 2, using the Roland-Morris Disability Questionnaire (RMDQ) score [22]. Amoxicillin-treated groups had a mean difference of −1.6 (95% CI: −3.1 to 0, *p* = 0.04), with secondary analysis of MC1 showing −2.3 (−4.2 to −0.4, *p* = 0.02) and MC2s showing −0.1 (−2.7 to 2.6, *p* = 0.95). Although the statistically significant findings in the MC1 group did not reach the minimally clinically important difference in RMDQ score of 4, the authors do not support the use of antibiotics in this cohort [22]. Kristoffersone et al. categorized patients with LBP and MCs 1 or 2 through STIR effect modifications predefined as STIR 1/2/3 (adjusted by volume, height, and maximum intensity), with higher intensities highlighting greater levels of edema [23]. The STIR3 group and STIR volume greater than 25% of the vertebral body both had greater than 4 RMDQ points, indicating clinically important improvements. The STIR3 group had a greater effect of Amoxicillin at −5.1 RMDQ (*p* = 0.008) compared to STIR1 and STIR2 at −2.3 and −0.1 RMDQ, respectively. Furthermore, the authors state that the STIR3 results with the abundant edema seen may reflect a low-grade discitis, and the treatment course mirrored that of *C. acnes* discitis. These results warrant further evaluation of biological markers of infection in relation to MC-related edema and to clarify STIR findings [23]. Additionally, Heggli et al. noted evidence for two different subtypes of MC1: (1) an intradiscal high genome copy number (GCN) *C. anes* subtype secondary to innate immune system activation and (2) an intradiscal low GCN *C. acnes* subtype secondary to adaptive immune system activation. This suggests the existence of a potential bacterial and autoimmune MC1 subtype. However, the current literature has contradictory conclusions, and the fluctuating focus between different types of MC creates greater confusion for clinicians [19].

**Table 2:** Features of Modic changes compared to other endplate abnormalities

Endplate abnormality	Typical location	MRI characteristics	Disc involvement	Distinguishing features
Modic changes (MCs)	Subchondral marrow adjacent to vertebral endplates	MC1: T1W hypointense, T2W hyperintense MC2: T1W hyperintense, T2W iso/hyperintense MC3: T1W/T2W hypointense	Disc degeneration without enhancement	Primarily at the endplates without paraspinal or soft tissue involvement
Discitis/osteomyelitis	Endplates and disc	T1W hypointense, T2W hyperintense (enhancement)	T2W disc hyperintensity and enhancement	Paraspinal soft tissue involvement with endplate degeneration, systemic infection symptoms
Schmorl’s nodes	Focal endplate change	Marrow edema can resemble MC1	Focal disc herniation into the vertebral body	Defined endplate defect that is localized and not diffuse
Compression fracture	Vertebral body adjacent to the endplate	T1W hypointense, T2W hyperintense with marrow edema	Not primarily with disc involvement	Fracture line, wedge deformity, and loss of vertebral height
Degenerative Sclerosis	Subchondral bone	T1W/T2W hypointense can resemble MC3	Disc involvement, but may be preserved	Usually chronic and diffuse without preceding inflammatory changes
Neoplasm	Vertebral body	T1W hypointense/T2W hyperintense (enhancement), but can be variable	Disc may be preserved	Often crosses multiple levels with possible soft tissue involvement

For the autoimmune etiology, Dudli et al. implanted intervertebral disc micro pellets into rat tail bone marrow, which led to T-cell infiltrate and MC1-like signal changes observed on MRI [17]. Dudli et al. also biopsied patients’ vertebrae who were undergoing lumbar spinal fusion to observe the histology and immunohistochemistry [5]. MC1, compared to MC0 (normal endplate), contained greater levels of connective tissue, edema, inflammatory infiltrates, and CD90 + cells, which are correlated with inflammatory-fibrotic changes. Similarly, MC2s had greater edema and CD90 + cells compared to MC0 [5]. Greater levels of connective tissue also correlated with the severity of LBP observed. The inflammatory-fibrotic mechanism is postulated to result from the accumulated structural endplate damage and resulting increased proliferation of bone marrow stromal cells (BMSCs) to repair the damage, leading to a fibrotic change [5]. Other mechanisms proposed have been direct damage from the release of lactate dehydrogenase (LDH) from damaged cells or pro-inflammatory changes in the disc [5]. However, the authors note that their included patients who required surgery may not be representative of other patients with MC, as most patients with CLBP do not require surgery [5].

The complement system’s involvement with MCs is not currently fully understood; however, emerging evidence suggests an important role in the inflammatory process. Specifically, MC1, characterized by bone marrow edema and increased vascularity, can drive complement recruitment and production [19]. These factors likely work alongside other etiologies in which a response of immunogenicity and bacterial defence mechanisms can drive autoantibody production, neutrophil activation, and osteoclast formation [19]. Heggli et al. posit that C6 deficiency is protective against osteoarthritis and bone degeneration; it is further plausible that it would be protective against MCs, although further research in this area is warranted [19]. The complement system is also involved in the recruitment of osteoclasts and building initial immune response cascades, yet it is still understudied and requires further research.

Genetic factor analysis also found links between MCs, Vitamin D receptor variants (VDR), and specific metalloproteinases in addition to connections between degenerative disc disease (DDD) and VDR variants [16]. The involvement of different genetic factors is very likely, although the specific genes involved and their role in these processes are not well understood.

## Risk factors and prevalence

Risk factors for MCs include male sex, age, severe diabetes mellitus, genetic factors, spinal deformities, disc displacements, and continuous load bearing [18]. Patients with DDD, disc degeneration, disc herniation, and endplate defects are more likely to have underlying MCs [18]. There is a wide variation in the prevalence of MCs among asymptomatic individuals, reported to be around 0.5–47.1% [16]. MCs are most commonly (74.5%) found on adjacent caudal and cranial vertebral endplates in the L4/5 and L5/S1 regions but can also be found in isolation [24]. Concerning the distribution of MCs within the endplate, 66% of the MCs identified involved the majority of the AP diameter of the vertebral endplate, and 17.5% involved the full transverse diameter [25]. The risk factors for MCs found in lower lumbar regions (L4/L5) are different from those of the upper regions (L1–L3). Disc displacement and degeneration are the main factors that predispose individuals to MCs with upper lumbar involvement [26]. Previous lumbar injuries, smoking, obesity, aging (with 4% increased odds of finding MCs each year), Schmorl’s nodes, demographics, and lifestyle factors have a more significant role in the lower lumbar regions [26]. Increases in MC size and intensity are associated with increased degenerative changes and other endplate defects such as disc herniation [14]. Different types of MCs often exist together, and varying conditions may predispose a person to one type or precipitate conversion from one type of MC to another [14]. Approximately 20% of MCs exist as mixed MC1 and MC2 lesions, possibly suggesting that these lesions occur as part of a singular pathological process that may also occur independently [24].

## Association with VBP

MCs 1, 2, and 3 were first identified for their link with discogenic back pain in 1988 after Modic et al. observed a consistent association between vertebral bone marrow signal intensity change and degenerative disc disease [27]. More recent studies increasingly investigated MCs for their potential link to VBP, which originates from the vertebral body rather than the disc itself [17]. Additionally, the presence of any type of MC in the lumbar region, as well as the number of endplates, was statistically associated with greater age (*p* < 0.001) [25]. The finding that MCs relate to age supports the degenerative nature of MCs and highlights similarities between disc degeneration and MCs [25]. Consistent with these findings, Matsumoto et al. found similar rates of MCs in patients 11 years following whiplash injuries as their control subjects [28].

A meta-analysis performed by Brinjikji et al. found an odds ratio (OR) of 4.01 (*p* = 0.04) for the presence of MC1 and CLBP, while only 3% of asymptomatic subjects exhibited MC1 [29]. Additionally, in a prospective MRI study on patients with CLBP and large MC1s, the increase in LBP was associated with bony endplate deformities, in addition to a decrease in disc height [30]. This study suggested that the source of non-specific LBP may be related to the abundance of nociceptive receptors in MCs and body endplate defects [30]. Supporting this hypothesis, a meta-analysis of 11,027 subjects demonstrated significant associations between back pain and specific endplate defects, including erosion (OR 2.69), sclerosis (OR 1.97), and Schmorl’s nodes (OR 1.63) [31]. These findings are consistent with cadaveric evidence showing strong correlations between endplate erosion, back pain, and adjacent disc degeneration [32]. Furthermore, Zehra et al. found that larger endplate defects were more commonly associated with the presence of MCs and disc degeneration, supporting the idea that endplate defects are structural abnormalities that generate lower back pain [33]. The study also emphasized the importance of assessing endplate defects using a multidimensional approach, considering both the size and shape of defects [33].

Research has shown increasing evidence that supports the hypothesis that pain decreases as MC1s convert to MC2s [30]. This hypothesis is supported by a retrospective study of 2457 lumbar spine discograms and prediscogram MR images, which found that MC1 changes on MRI strongly indicate a likely source of pain, whereas MC2 changes had a lower positive predictive value for a positive discogram [34]. A longitudinal study of MC1 endplate changes correlated the changes with pain induced by discography, finding a close relationship between MC1 changes and pain and no relationship with MC2 changes [35]. Additionally, a 2014 prospective nested cohort study demonstrated that patients with MC1s at baseline had a significantly higher risk of no improvement in pain and function compared to those with MC2 changes [36]. However, in a large population-based study, Teraguchi et al. reported that MC 1/2 were associated with LBP, with an OR of 3.26 (*p* < 0.0001) compared to types 1, 2, 2/3, and 3, which were not significant [15]. Additionally, MC1 and MC2 changes at the L2/3 and L4/5 levels were significantly correlated with the presence of LBP [15]. These findings support the association of both MC1 and MC2 with LBP, although evidence suggests that MC1 changes may have a stronger correlation.

While MCs have been increasingly implicated as a VBP marker, the differentiation between VBP and discogenic sources is clinically important.

## BVN and BVN ablation

The anatomical correlation of the BVN to the vertebral endplates explains the “endplate-driven” model of discovertebral pain, now named vertebrogenic pain [11]. Vertebral endplate pain has been observed to present with significant functional impairment as well as debilitating pain located in the midline region of the lumbar spine [37]. Conservative treatment for VBP, such as oral anesthetics, opioids, and therapeutic exercises, has limited efficacy [37].

BVN ablation is a minimally invasive outpatient procedure that was FDA-approved in 2016 as a treatment for targeting the origin of the pain from damaged vertebral endplates by interrupting the nociceptive signal from the BVN [37, 38]. A systematic review of 11 studies revealed that BVN radiofrequency ablation (RFA) had clinically significant benefits in a reduction in pain and disability, opioid reduction, and improvement in quality of life in patients with MC1 and MC2 changes [1]. Studies indicate that bone marrow intensity changes (BMIC) associated with MC1 and MC2 are crucial for predicting treatment success with BVN RFA because these changes reflect the severity of endplate damage and associated pain [39]. All other endplate, intervertebral disc, facet joint, spinal segment alignment, foramina, lateral recess, central canal MRI findings, and anterior/posterior column degenerative findings were not associated with clinically important predictive models for BVN RFA success [39].

Fischgrund et al. followed up at 5 years post-ablation and found a sustained clinical improvement in function using patient-reported outcomes of the Oswestry Disability Index (ODI), supporting the use of BVA ablation for positive long-term results [40]. Similarly, at 12 months, Smuck et al. found a significant improvement in ODI, visual analog scale (VAS) pain, and quality of life outcomes (*p* < 0.001 for all) with BVN RFA compared to standard care, which were sustained at two years [3, 41]. Macadaeg et al. at 12-month follow-up also found significant improvements in ODI, VAS, SF-36, and EQ-5D-5L scores (*p* < 0.001 for all) in patients with MC1/MC2 [42]. The current literature outlines strong recommendations for BVN ablation in patients with chronic axial LBP of greater than 6 months, unresponsiveness to 6 months of conservative treatment, and diagnostic evidence of MCs suggesting vertebral endplate damage [2, 37, 43].

Interestingly, McCormick et al. suggest that while MCs are strongly associated with vertebrogenic pain, the treatment success with BVN RFA is not solely determined by the degree of MC damage or the presence of endplate defects [39]. They also found that patients with smaller volume MC, non-centrally located MC, and those with < 25% involvement of the endplate responded similarly to BVN RFA treatment as those with large volume MCs [39]. These findings highlight the complexity of using MCs alone to predict BVN RFA success rates and show that BVN RFA can be effective regardless of the size of the MCs. Overall, BVN RFA has promising findings, but further high-quality studies are required to elucidate its long-term effects and efficacy across different patient populations.

## Intradiscal injection therapy

While steroid injections have been widely explored as a common treatment for spinal pain caused by disk herniation [44], there has been limited research addressing their efficacy in treating VBP. Two widely known forms of spinal steroid injections are intradiscal injections and epidural injections, with epidural injections having transforaminal, interlaminar, and caudal approaches [45]. It has been hypothesized that intradiscal steroid injections can be a method of providing short-term pain relief for patients with anterior column pain, which is defined as pain originating from the intervertebral disc (discogenic) or vertebral endplates (vertebrogenic) [46]. Corticosteroids have been commonly used in chronic pain procedures due to their anti-inflammatory, immunosuppressive, vasoconstrictive, and antiproliferative effects [44]. In a retrospective review of 66 patients with CLBP and MCs who underwent intradiscal injection, there was a statistically significant difference in the pain scores between pre-injection (7.31 ± 2.23) and follow-up evaluation (3.96 ± 2.50), as well as a significant difference between pre-injection and maximum relief scores [46]. In patients with DDD and MC1 end-plate inflammatory changes who underwent epidural steroid injections, patients with MCs had a greater improvement in Oswestry Disability Index (ODI) and pain diagram (PD) scores in the first 6 months after injection than those without the endplate changes [47]. Furthermore, the study found that additional intradiscal injections led to significant improvement in those with MCs in all outcome scales [47]. Given the association of MC1 changes to VBP [29], these findings highlight the potential pain-relieving effects of both epidural and intradiscal corticosteroid injections for patients with DDD and MC1s.

Intradiscal injection is generally safe with an overall complication rate of 0.47% (32/6843) pooled across six trials [48]. However, the adverse effects of discitis, disc collapse, and transient impairment of sensitivity in the ipsilateral limb have been reported [49]. The greater concern is the potential contribution to intervertebral disc degeneration. Caragee et al. raised concerns about accelerating disc degeneration following disc puncture and injection during discography, even with small-bore needles and limited-pressure injections [48]. Thus, the authors conclude that intradiscal therapies such as steroids, stem-cell therapy, and platelet-rich plasma should weigh the benefit against the potential risk of further degeneration [48]. Needle punctures theoretically increase cell death, metabolic dysfunction, disruption of annulus fibrosis integrity, and depressurization of the nucleus pulposus [49]. However, a 2020 narrative review of 6843 patients noted that there were two cases of a collapsed disc following intradiscal injection [50]. Notably, a 2024 study by Ohtonari et al. using intradiscal injection of Condoliase found that Pfirrmann disc grade progressed in 20/41 patients (48.8%) at three months, but 8/20 (40%) patients recovered [51]. However, this was only observed with Pfirrmann grades IV to III at nine months. However, one in three (33.3%) patients progressed from grade II to grade IV at nine months [51]. The Pfirrmann grade intervertebral discs on MRI based on disc structure, signal intensity, nucleus pulposus and annulus fibrosus distinction, and disc height. This system is graded from I (homogenous disc, bright nucleus with clear annulus distinction) to V (collapsed disc, inhomogeneous signal, loss of nucleus-annulus distinction) [52]. Intermediate grades (II-IV) represent a progressive decrease in signal intensity, structure, and height [52]. Thus, despite the clinical improvements that can be seen in intradiscal therapies, these risks must be taken into consideration. Alternative methods to mechanical puncture are being developed to minimize the mechanical disruption to the disc, which include hydrogel-based material, nanoparticle-based material, and stimuli-responsive delivery systems [52, 53]. Overall, the role of intradiscal injection therapies may be beneficial, but these effects are often short-lived, with a wide variety in study design and agents used. Thus, firm conclusions on their overall role in managing MC and VBP are unable to be drawn.

## Biomarkers

Preliminary proof of concept study by Aboushaala et al. discovered several statistically significant blood biomarkers in patients with MCs: elevated C-C Motif Chemokine Ligand 5 (*p* = 0.0006), decreased Macrophage Migration Inhibitory Factor (*p* = 0.009) [54]. Other biomarkers that had potential relevance included C-X-C Motif Chemokine Ligand 5 (*p* = 0.052), Pentraxin 3 (*p* = 0.06), and Galectin-3 (*p* = 0.07) [54]. However, Schulze et al. conducted a proteomics approach using the Data-Independent Acquisition-Based (DIA) Sequential Window Acquisition of all Theoretical Fragment Ions Mass Spectrometry (SWATH-MS) and an ELISA-based method called V-PLEX and did not find any association between serum biomarkers and MCs [55]. Dudli et al. found that biomarkers contributing to type III and type IV collagen degradation/formation correlated with the presence of MCs (*p* = 0.06 to 0.08) [56].

## Imaging

Current guidelines for LBP recommend against imaging unless red-flag symptoms or neurologic signs are present. However, for patients with VBP, MRI is required for diagnosis, and future guidelines may lead to increased use of imaging for these subsets of patients [57]. MRI is the most sensitive modality to visualize endplates and can detect abnormalities, breaks, disruptions, or other discontinuities of endplate morphology. Radiologic modalities other than MRI have been suggested for diagnosing VBP and detecting MCs. Computed tomography (CT) can identify endplate degenerative defects and be a useful tool for patients with contraindications or other barriers to MRI access [57].

Single photon emission computed tomography (SPECT), a bone scan with radiotracer overlaid on a three-dimensional CT image, has been proposed for this purpose due to inflamed endplates theoretically having greater uptake of the radiotracer. Jarvinen et al. found via SPECT that MC1s had increased radiotracer uptake in MC1s, which reflects the increased metabolic activity and increased bone turnover [58].

**Fig. 3:**
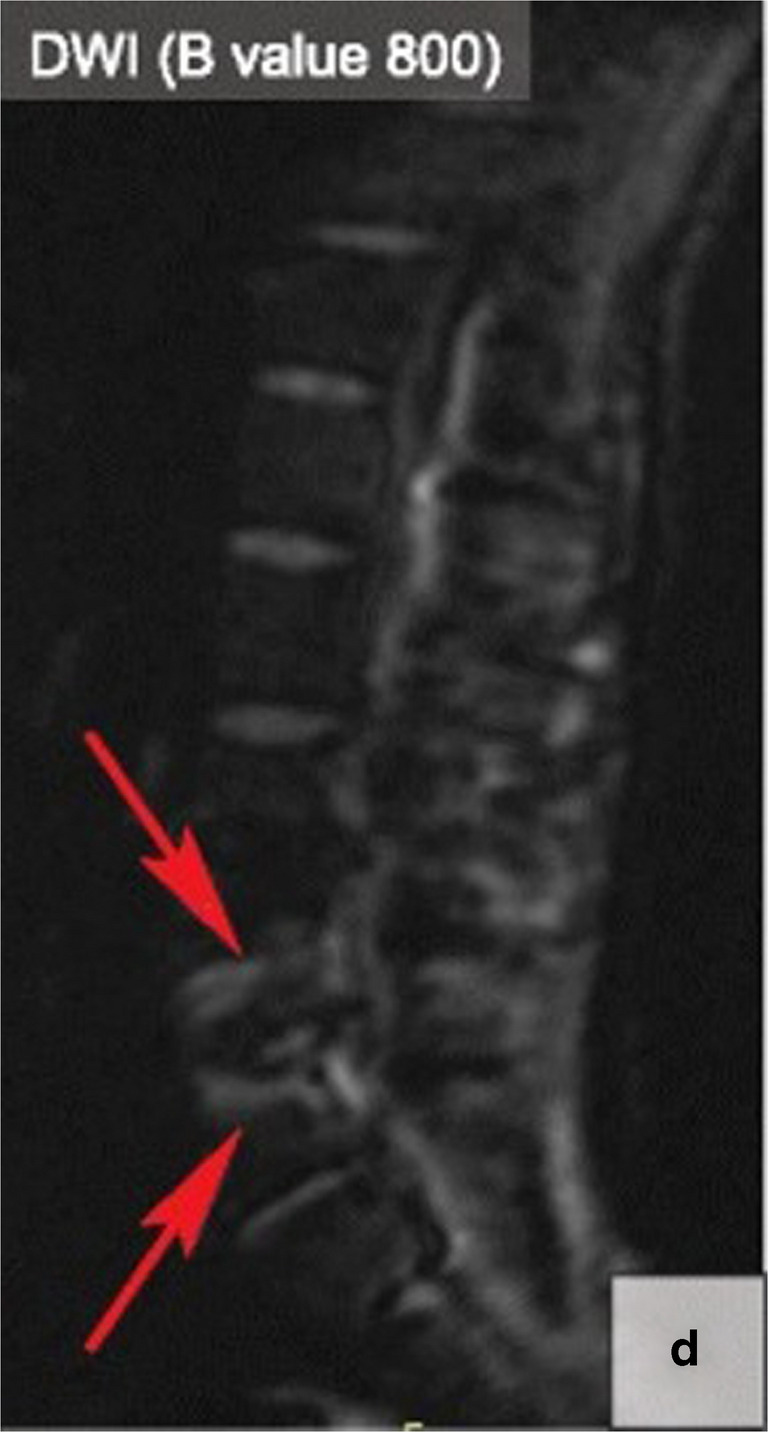
Diffusion-weighted imaging of the “claw” sign, representing distinct paired hyperintensities, indicating Modic type 1 changes. Adapted from Kushchayev et al., panel D, licensed under CC BY-4.0 [60]

**Fig. 4:**
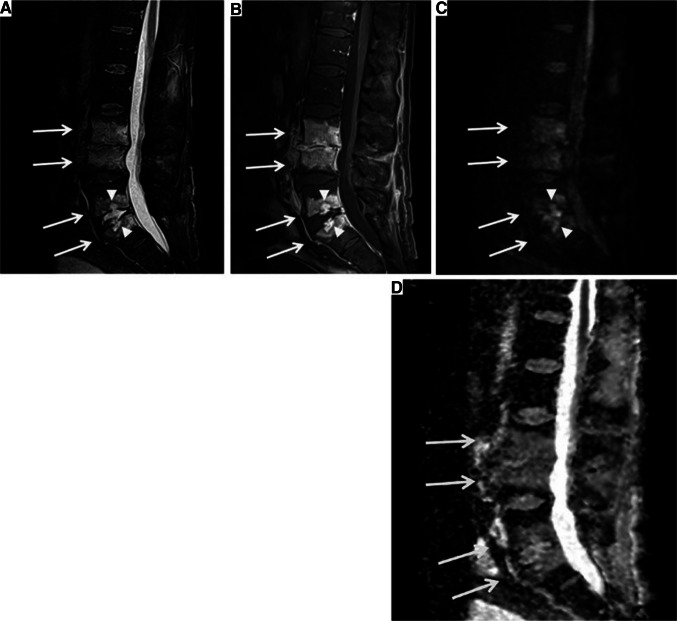
Diffusion-weighted imaging of the “reverse claw” sign in panel D, representing diffuse ill-defined hyperintensity, suggestive of discitis/osteomyelitis. Reproduced from Weaver et al., licensed under CC BY-NC-ND 4.0 [61]

Diffusion-weighted imaging (DWI), a rapid MRI sequence that approximates microvascular perfusion and diffusion of water molecules, can be a useful tool for differentiating MC1 versus a spinal infection with decreased and increased signals, respectively [59]. The “claw” and “reverse claw” signs on DWI are important, especially if clinical and laboratory findings are indeterminate for MC versus discitis/osteomyelitis [59]. The “claw” sign represents a well-defined paired hyperintensity within the vertebral bodies adjacent to a disc on sagittal DWI, which is consistent with degenerative changes (MC1 signal changes) (Fig. 3). “Reverse claw” or absence of the “claw” sign, on the other hand, is demonstrated by ill-defined margins of diffuse hyperintensity in the affected vertebral bodies, suggesting discitis/osteomyelitis instead (Fig. 4) [59].

Water-fat MRI spatially assesses bone marrow fat (BMF), which has been shown to correlate with histological findings. Magnetic resonance spectroscopy (MRS) also measures fat content in the vertebral body and has good equivalence with water-fat MRI [59]. These two quantitative imaging techniques can theoretically quantitatively and continuously measure endplate marrow injury, potentially monitoring disease progression and subsequent treatment [59]. Other advanced imaging methods, such as ultra-short echo time imaging and quantitative MRI, can increase the accuracy of detecting endplate changes and MCs [57].

An important consideration when interpreting imaging for endplate changes is differentiating between MC, discitis/infection, Schmorl’s nodes, compression fractures, degenerative sclerosis, and neoplasms (Table 2). Considerations of location, marrow signal pattern, level of disc involvement, and any associated soft tissue changes are essential to diagnosis.

## Implications

The improved understanding of VBP has led to a paradigm shift in the approach to diagnosing and managing LBP. The increased awareness of VBP has led to a greater focus on vertebral body endplate changes, which can be visualized on histology or with MCs on MRI [62]. A retrospective study of patients at an academic spine center found strong agreement on identifying LBP and MCs on imaging, but episodic back pain had poor sensitivity (20%) and limited diagnostic value (confidence 55%) for detecting MC1 or MC2 changes [51]. In contrast, severe episodic back pain has been strongly associated with a discogenic etiology (58% sensitivity, 88% specificity, and 95% diagnostic confidence) [63].

Clarifying the pathomechanisms can identify risk factors and help develop pathomechanism-specific treatments. MC1s have poorer long-term outcomes and response to conservative management compared to MC2s, likely due to the elevated levels of inflammatory mediators [24]. MC1 is also correlated with constant LBP pain and stiffness compared to its type 2 counterparts. Furthermore, mixed type ½ and ⅔ have been observed as well as an interconvertible process (Fig. 1) [64]. Principal component analysis (PCA) of MC1 biopsies found significant heterogeneity among these samples, which indicates the presence of subtypes, postulated from different histories or etiologies of the MC lesions [5]. The advancement of artificial intelligence and deep learning modalities has shown strong success in detecting and classifying MCs with strong agreement with physician diagnoses [65–67].

## Future directions

However, the magnitude of MCs’ effect on pain and disability may vary depending on the specific type of MC characteristics and personal psychosocial factors, as evidenced by patients who do not benefit from BVN ablation. MCs can present asymptomatically, symptomatically with pain and disability, or pain with provocative discography [68]. It is not well understood yet whether symptomatic and asymptomatic MCs are different. Among the chronic LBP cohorts, MCs are prevalent in 43% of patients versus 6% in asymptomatic patients [69]. It is currently unknown whether asymptomatic MCs will progress into painful MCs and how prevalent BVN involvement in these patients is. Thus, further research into the pathogenesis and etiologies behind symptomatic and nonsymptomatic patients is required.

Czaplewski et al. propose that studies should focus on homogeneous patient subgroups, including those that are difficult to treat, such as persistent nonspecific chronic LBP. This may also assist in a better understanding of these underlying pathologies and potentially provide more options for treatment [70]. Herlin et al. pooled together studies that grouped all modic change types, those that did not separate acute versus chronic LBP, and studies including patients with comorbidities such as herniation or scoliosis [71]. Though the authors acknowledge the heterogeneity in their review, Czaplewski et al. note that inappropriate citation and the conclusion that CLBP is not associated with MCs can delay the development of future therapies and possibly insurance coverage [69].

BVN ablation is an effective intervention, but it is an invasive procedure. However, it does indicate that MC1 and MC2 changes may be contributors to pain and disability. Future treatments may focus on targeting the causes of MCs, such as inflammation or MC sequelae, such as bone remodeling. Other treatments for VBP aside from BVN ablation include extraosseous epiduroscopic BVN laser ablation or bipolar radiofrequency ablation, intraosseous plasma-rich growth factor, intraosseous bioresorbable cement injection, and endoscopic disc debridement surgery [11].

Future studies should assess the correlation of MCs, along with their different characterizations, and patient pain and disability scores. Longer-term studies to evaluate the natural progression and interchangeability of MCs and the correlation with symptom development over time are also needed. A deeper understanding of the pathomechanisms and pathophysiological developments underlying MCs can help identify novel therapeutic targets and effective treatments for CLBP with MCs. The continued advancement of clinical, imaging, or other unexplored characteristics of VBP can enable further progression of the understanding of the role of MCs in this process.

## Conclusion

MC, especially MC1, has emerged as a potential MRI indicator for VBP, which represents a subtype of CLBP generated from vertebral endplate and body pathology. The pathophysiology of MCs is broad, multifactorial, and not fully understood. However, current hypotheses encompass mechanical disruptions, inflammation, bacterial, and autoimmune etiologies and have been increasingly linked to nociceptive signaling from the BVN. Despite the promising findings of BVN ablation, patient selection may be the crucial factor for this procedure’s success. Thus, diagnostic and criteria standardization is a major gap for further research to produce more consistent therapeutic outcomes. Future directions with biomarkers, advanced emerging imaging techniques, and clinical translation are required to refine the clinical role of MCs in diagnosing and managing VBP.

## Data Availability

Data sharing is not applicable to this article as no datasets were generated or analyzed during the current study.

## Abbreviations

*CLBP*: Chronic lower back pain
*LBP*: Low back pain
*BVN*: Basivertebral nerve
*VBP*: Vertebrogenic back pain
*MCs*: Modic changes
*MRI*: Magnetic resonance imaging
*MC1*: Type 1 MCs
*STIR*: Short tau inversion recovery
*MC2*: Type 2 MCs
*MC3*: Type 3 MCs
*PGP 9.5*: Protein gene product 9.5
*T1W*: T1-weighted
*T2W*: T2-weighted
*NP*: Nucleus pulposus
*C. acnes*: Cutibacterium acnes
*RMDQ*: Roland-Morris Disability Questionnaire
*GCN*: Genome copy number
*BMSCs*: Bone marrow stromal cells
*LDH*: Lactate dehydrogenase
*VDR*: Vitamin D receptor variants
*DDD*: Degenerative disc disease
*OR*: Odds ratio
*RFA*: Radiofrequency ablation
*BMIC*: Bone marrow intensity changes
*ODI*: Oswestry Disability Index
*PD*: Pain diagram
*DIA*: Data-independent acquisition-based
*SWATH-MS*: Sequential window acquisition of all theoretical fragment ions mass spectrometry
*CT*: Computed tomography
*SPECT*: Single photon emission computed tomography
*DWI*: Diffusion-weighted imaging
*BMF*: Bone marrow fat
*MRS*: Magnetic resonance spectroscopy
*PCA*: Principle component analysis
